# Development of a Thin-Film Sensor for In Situ Measurement of the Temperature Rise in Rolling Contacts with Fluid Film and Mixed Lubrication

**DOI:** 10.3390/s21206787

**Published:** 2021-10-13

**Authors:** Stephan Emmrich, Marcel Plogmeyer, Dirk Bartel, Christoph Herrmann

**Affiliations:** 1 Chair of Machine Elements and Tribology, Faculty of Mechanical Engineering, Otto von Guericke University Magdeburg, Universitaetsplatz 2, 39106 Magdeburg, Germany; dirk.bartel@ovgu.de; 2 Fraunhofer Institute for Surface Engineering and Thin Films IST, Bienroder Weg 54 E, 38108 Braunschweig, Germany; marcel.plogmeyer@ist.fraunhofer.de (M.P.); c.herrmann@tu-braunschweig.de (C.H.)

**Keywords:** mixed friction, fluid friction, thin-film sensor, rolling contacts, high pressure

## Abstract

The following study presents an in situ sensor system which can measure the temperature change of rolling contacts for heavy duty during fluid as well as mixed friction. This thin-film sensor was optimized with regard to its size, spatial resolution, and wear resistance. Extensive tests were carried out with a two-disk test rig and the data of the temperature change were presented. The results show the complex processes within a rolling contact and the strongly interaction of pressure, friction, and temperature development within the contact zone. Due to the detailed sensor and disk characterization, the data are suitable for comparing calculation methods.

## 1. Introduction

The optimization of friction and wear for rolling contacts is a main issue in the design of machine elements, such as bearings or gears, regarding more energy efficiency and saving material resources. Historically, the optimization of rolling contacts is carried out by experimental design and a large number of tests. The contact temperature is one important issue while concerning rolling contacts. With a certain distance of few millimeters to the contact area the rise of temperature and the contact forces are easy to measure and can be recorded with simple devices and test rigs. However, these measurements and evaluations only indicate indirectly the processes within the contact zone. The in situ measurement of temperature rise, contact pressure and lubrication film thickness using a thin-film sensor provides an exact insight view of rolling contacts. Nevertheless, the in situ measurement within fluid friction (FL) is already a big challenge even though simple test rigs are used. If the friction type changes from fluid to mixed friction (MF), the in situ measurements will be far more challenging. Mixed friction is of particular interest when real tribological systems such as gears or rolling bearings are considered. The measured data of these in situ measurements can not only be applied to optimize machine elements but also to carry out comparative measurements with modern thermo-elastohydrodynamic lubrication (TEHL) calculation models to refine these models. This leads inevitably to an extended understanding of rolling contacts.

In order to measure the processes of a tribological contact within a machine element under real conditions, thin-film sensors have become an established tool over several decades. The first use of thin-film sensors to measure the thickness of a lubrication film in a two-disk contact goes back to Crook [1]. He used chrome as a capacitive sensor under fluid friction. The first use of thin-film sensors for pressure and temperature measurement in a two-disk contact under fluid friction was performed by Orcutt [2]. He modified Crooks’ test setup by integrating additional temperature and pressure sensors into the running surface of the disks. Platinum thin-film resistors were used to measure the temperature in the contact area. The pressure (maximum at 278 MPa) was measured using thin-film resistors made of manganin. Köhler investigated the pressure and temperature change in a two-disk contact [3]. He also integrated thin-film sensors in real machine elements like in rolling bearings and in journal bearings. The sensors were made of manganin, titanium, cupper-nickel, or a nickel-chrome alloy. A silicon or metal oxide layer was used as an insulating layer. The severe conditions of mixed friction were avoided during the tests, however. Safa et al. used thin-film sensors to measure pressure, temperature, and lubricant film thickness in a ball-on-disk setup as well as in a rolling bearing under fluid friction. The thin-film resistors consisted of manganin for pressure and titanium for temperature measurement. For the capacitive measurement of the film thickness, they used a thin-film electrode. A thin aluminum oxide layer insulated the sensors from the counter disk or the rolling bearing outer ring. In general, the results of the measured lubrication film thickness confirmed the theoretical prediction of Dowson and Higginson [4,5]. In addition, Simon investigated the limits of various thin-film sensors for measuring pressure and lubricant film thickness in a two-disk contact under fluid lubrication [6]. His film thickness measurements with contact pressure up to 1300 MPa showed accordance with a calculation tool of Oster [7]. Baumann then compensated pressure measurement errors with a double layered sensor by varying the layer thicknesses of manganin and titanium [8,9]. The following pressure and temperature measurements were carried out in a concentrated elastohydrodynamic lubrication (EHL) contact under fluid friction while using silicon oxide as insulating layer. His focus was on pure rolling and sliding with four different reference oils but also set first steps into mixed friction and compared his experimental pressure and temperature data with published calculation data. Peeken et al. used aluminum oxide as an insulating layer and titanium or manganin as sensor materials on gear tooth flanks [10]. He measured the temperature and pressure as well as the lubricant film thickness by varying the surface texture. The experiments were attended with numerical calculation. Kagerer, together with Peeken, was one of the first to apply thin-film sensors on tooth flanks [11,12]. He used sputtered thin film resistors made of manganin, titan, and copper for his tribological investigations of pressure, temperature, and film thickness on disks and tooth flanks. Aluminum oxide (Al_2_O_3_) layers were used both as insulating layer and as top layer for measurements of up to 1600 MPa. However, the investigations of the tooth flank contacts were mainly carried out without a protective top layer by means of fluid friction. In addition to his error analysis and error correction he compared his measurement data with the calculation tool of Oster [7]. Tychsen also conducted tribological measurements on tooth flanks with a thin-film sensor [13]. He used silicon oxide as an insulating layer to the gear wheel and titan for film thickness measurements. The settings were limited by 1000 MPa pressure and fluid friction. With his work he followed also the efforts of comparing TEHL data with real experiments. Mihara et al. integrated a pressure sensor into the main plane bearing of combustion engines based on preliminary work of Köhler [3,14]. Manganin as resistor and silicon oxide as an insulating and protection layer were used as thin-film sensor. The temperature rise was determined with thermocouples. Low pressure ensured suitable measurements. Kühl and Holland followed the work of Tychsen to integrate a thin-film sensor on a tooth flank [13,15,16]. The well-known silicon oxide was used as an insulating layer. This time the pressure and temperature sensors were designed as thin-film sensor made of chrome and titanium. The measurements were carried out under fluid friction and a pressure of up to 1450 MPa. Therefore, a protective top layer was not required.

Within his tribological investigations on ceramic steel disks Sperrfechter developed an iterative algorithm for an error compensation of combined pressure and temperature sensors [17]. This algorithm represented an alternative to double-layer transducers from Baumann and later from Kühl [8,9,15]. The lubrication gap height was measured capacitively between a thin-film electrode and a metallized ceramic disk. Thereby he took the temperature and pressure dependence of the dielectric constant of the oil into account. He investigated the influence of various ceramic materials (Al_2_O_3_, ZrO_2_, AlN) and steel as base material. As insulating and wear protection layer he used Al_2_O_3_. The sensors were operated in a two-disk setup under fluid friction with a pressure of 1300 MPa. Dauber investigated EHL in steal-ceramic contacts under pure rolling with consideration of slight sliding, which are typical for rolling bearings [18]. He focused his investigations on the generation of friction, in respect to the contact parameters temperature, pressure, and lubricant film thickness. For the error compensation he optimized the determination of the sensor position, which is significant due to the high temperature and pressure gradients. In addition, he considered the non-Newtonian oil behavior for calculating the lubricant gap height. The tests were carried out at fluid friction and pressures of up to approximate 900 MPa. In his investigations on the influence of the surface structure on the pressure distribution in two-disk contacts Kreil used Al_2_O_3_ as insulating layer [19]. He stated a period of more than two hours for the thin-film sensor lifetime. The sensor was designed without a protection layer, therefore he avoided mixed friction. Miyata focused his work on continuously variable transmission and designed for this purpose a test setup on a two-disk test rig to measure the temperature rise with thin-film sensors [20]. His experimental work was based on Simon, Kagerer, and Kreil [6,11,12,19]. Mayer measured the pressure and temperature change by means of thin-film sensor technology in a two-disk contact [21]. Instead of an insulating layer he used a disk made of Al_2_O_3_. Therefore, the sensor layer was coated directly on the surface. Due to the lack of a protection layer, measurements were only conducted under wear-free fluid friction with focus on the surface texture influence. Ebner et al. used platinum sensors on ceramic disks to study the influence of thermal insulation effects on temperature rise in rolling contacts [22]. To analyze the measured data, he used a TEHL model of Lohner and achieved with his consideration an accordance of simulation and experiment for fluid friction up to 1000 MPa pressure.

With the current state of thin-film sensor technology the relevant contact parameters pressure, temperature, and lubrication film thickness can only be measured under wear-free fluid friction. Moreover, in recent years, the experimental pressure settings were significantly lower than in many technical rolling contacts. Hence, as a first step, a thin-film sensor was developed in this work to measure the temperature rise during mixed friction under high load up to 1750 MPa. The sensor system is considered on bearings and gears in a further step. Additionally, the sensor was mechanically and thermally characterized by means of the possibility of comparing the temperature measurements with TEHL calculations with consideration of real surfaces and insulating effects due to coatings.

## 2. Experimental Setup

### 2.1. Test Rig

**Figure 1:**
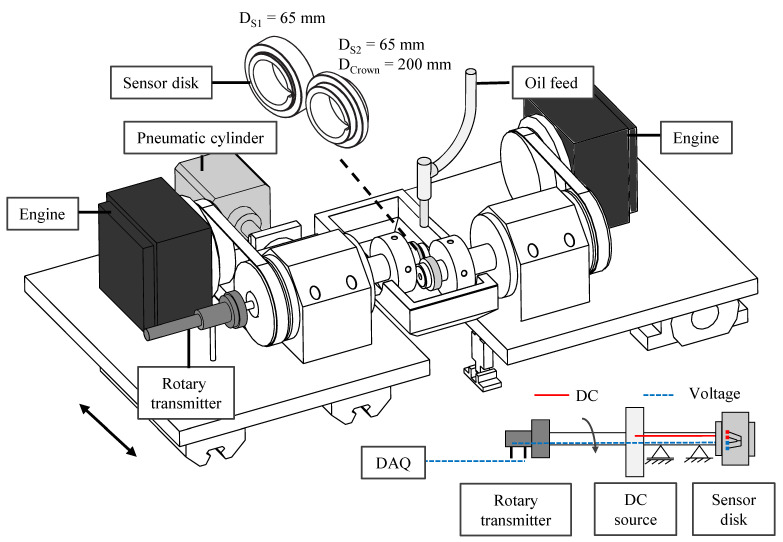
Two-disk test rig.

A two-disk test rig (Figure 1) was used to perform the measurements of the temperature rise in a rolling contact under high pressure and mixed friction. The sensor is applied to the surface of a cylindrical sensor disk. In order to avoid damage to the sensor connections, the second disk was designed thinner to exclude over-rolling. While pairing two cylindrical disks forms a line contact, a point contact results by pairing a cylindrical and a crowned disk. By reason of stress peaks occurring at the edge during line contact, a crowned combined with a cylindrical disk was favorized in this study. During the tests the rotating disks powered by two separate engines are pressed against each other with a pneumatic cylinder. Due to the elasticity of the steel disks a parabolic pressure distribution is formed p(x,y), Equation (1).

(1) $$p\left(x,y\right){=p}_{\max}\cdot \sqrt{1\;-{\left(\frac{x}{b}\right)}^{2}-{\left(\frac{y}{a}\right)}^{2}}$$

The maximum pressure p_max_ occurs in the contact area center and is calculated by Equation (2).

(2) $$p_{\max}=\frac{3}{2}\;\cdot \;\frac{F}{A_{\mathrm{Hertz}}}$$

The area A_Hertz_ of the elliptical contact surface is calculated using Equation (3).

(3) $$A_{\mathrm{Hertz}}=\pi \;\cdot \;a\;\cdot \;b$$

The semi-axes a and b are calculated as a function of the force F and the elasticity E_Red_ and curvature ρ of the disks with applying the correction factors c_a_ and c_b_, Equation (4).

(4) $${a=c}_{a}\cdot \sqrt{\frac{3\;\cdot \;F\;}{2\;\cdot {\;E}_{\mathrm{Red}}\cdot \sum \rho }}\;\mathrm{and}\;{b=c}_{b}\cdot \sqrt{\frac{3\;\cdot \;F\;}{2\;\cdot \;E_{\mathrm{Red}}\cdot \sum \rho }}$$

As the disks rotate, the rectified tangential surface velocities v_1_ and v_2_ of the two disks pump heated oil, supplied by an oil feed, in the gap and create a supporting lubricant film (Section 2.3). The sum of both surface velocities is calculated according to Equation (5).

(5) $$v_{\mathrm{Tot}}{=v}_{1}{\;+\;v}_{2}$$

The difference between the two tangential velocities is called sliding velocity v_Sli_, Equation (6).

(6) $$v_{\mathrm{Sli}}{\;=\;v}_{1}{\;-\;v}_{2}\;\mathrm{with}\;v_{1}\;\geq {\;v}_{2}$$

The slip S as the ratio of sliding speed to tangential velocity can be calculated according to Equation (7).

(7) $$S=\;(\frac{v_{1}{\;-\;v}_{2}}{v_{1}})\;$$

Sliding in conjunction with friction generates heat as energy dissipation and this leads to an increasing contact temperature. By means of the oil feed temperature T_Oil_ and the total speed, the height of the lubrication film and thus fluid or mixed friction can be set systematically.

The measurement of the contact temperature is based on the change in resistivity ΔR due to temperature change of a metallic sensor layer. For this purpose, multilayer thin-films consisting of an insulation layer, a structured sensor layer, and a wear protection layer were applied to cylindrical steel disks. The insulation layer prevents a short circuit to the basic steel material. In an intermediate step, the sensor layer is structured to build a sensor in four-wire technology. The wear protection layer in turn avoids material removal on the sensor layer during mixed friction and prevents a short circuit to the counter crowned steal disk. In contrast, Kühl recommended performing the measurements without a wear protection layer in terms of an insulation error [15]. To avoid short circuit, this insulation error can be considered by TEHL-simulation like described by Bobach and Beilicke [23,24].

The temperature dependent change in resistance ΔR_T_ of the sensor in contact is superimposed by a resistance change due to the piezoresistive effect and the deformation of the transducer. The relative resistance change can be approximately described as a linear dependence on the temperature and pressure change (Equation (8)) [16]. The four-wire technology enables the detection of the resistance change in the sensor structure only and hence the temperature change with a high spatial resolution. Two of the four wires are connected to a constant current source (here I_Const_ = 4 mA). The voltage drop U(t) and thus the change in resistance can be measured directly with the two remaining wires combined with the Ohm’s law, Equation (9). The spatial resolution improves with a higher ratio of contact length to sensor length, 2·a > L_Sensor_.

(8) $$\frac{\Delta R\left(t\right)\;}{R_{0}}\approx {\;\alpha }_{T}\cdot \Delta T\left(t\right)+\;\alpha_{P}\cdot \Delta p\left(t\right)$$

(9) $$U\left(t\right)=R\left(t\right)\;\cdot {\;I}_{\mathrm{Const}}$$

To measure the contact temperature, the two-disk test rig is extended with a constant current source, a mercury rotary transmitter, a sensor disk, a trigger, and a data acquisition unit. The voltage signals are digitized with a data acquisition box with a maximum sample rate of 2 MS/s and an absolute accuracy of 2.688 mV at a range of 20 V up to 0.313 mV at a range of 2 V (DAQ NI 6366).

### 2.2. Test Specimen

The geometrical and physical properties of the disks have a strong influence on the tribological contact conditions. Therefore, the disks were extensively characterized in advance to use these properties to set up an TEHL-model and to compare the experimental temperature data with TEHL simulation data in a further step. The macroscopic and microscopic geometry, the mechanical and thermal properties of the layers, as well as the temperature and pressure dependence of the sensors were considered.

Table 1 summarizes all important geometric parameters of the disks which have an influence on the tribological contact. The diameter of the disks was measured with a universal length measuring device (ULM 600.2, resolution ±1 µm). The crowning and the surface micro geometry were determined optically with a white-light interferometer (Veeko Wyko NT9100). An attempt was made to obtain a sufficiently large measuring field, but this was limited by the curvature of the disks in terms of the measuring range of the optics.

The sensor design requires a very smooth surface to avoid a short circuit due to roughness elevations between the substrate material and the sensor. Therefore, the disks carrying the sensor were polished. In contrast, the counter disks were designed to be rougher, since mixed friction conditions should be achieved during the experimental tests at technically relevant speeds. Mixed friction condition is a combination of fluid friction and solid friction. Under mixed friction, the contact forces are transmitted on the one hand through the supporting lubricant film and on the other hand through microcontacts (Section 2.3). The formation of the micro contacts and therefore the real contact area depends strongly on the roughness and their plastic and elastic properties. In addition to the real surfaces the elastic-plastic dependency of the surfaces was measured, Table 2.

Within the micro contacts the contact forces lead to high stress on each single contact point. This causes high stress up to material deformation. This in turn increases the real contact area. In order to take this into account for the design of the experimental testing but also for the simulation study, the indentation modulus E_IT_ and the Martens hardness HM were determined by means of the instrumented indentation test (H100 Fischer 1000 mN) using a Vickers diamond. Under increased load, the diamond penetrates a near-surface area. After reaching the maximal load, the diamond is released. As a result of this experiment, load displacement curves are obtained. These were used to determine the indentation modulus and universal hardness to characterize the elastic and plastic behavior of the surfaces, Table 2.

**Figure 2:**
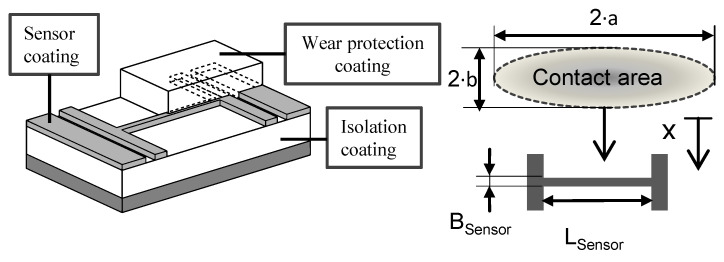
Sensor design.

The sensor not only consists of one but of three different layers, Figure 2. Starting from the steel surface, first an Al_2_O_3_ isolation layer was applied by physical vapor deposition (PVD) to avoid a short circuit with the steel material. This layer was applied with a thickness of 4–6 µm to ensure insulation of at least a few MΩ. Then a 0.2 µm thick chromium layer was coated and in a second step it was structured by photolithography and wet-chemical etching to obtain the sensor. On top, a hydrocarbon coating modified with silicon and oxygen (a-C:H:Si:O) with a thickness of 2 µm was applied by plasma-enhanced chemical vapor deposition (PACVD) to avoid a short circuit with the counter disk and to protect the sensor layer from constant material removal. Although these layers have just a thickness of a few micrometers, they have a significant influence on the heat flow. As a result, the frictional heat energy dissipates more slowly from the contact area and the temperature in the contact area is consequently higher than in a comparable steel-steel pairing [24].

In order to take this effect into account, the layer thicknesses were measured in a cross-section with a scanning electron microscope, after using the sensors. An ion etching process was used to expose the cross-section. The thermal conductivity of the sensor layers was determined using the 3-omega method. For this purpose, a similar sensor structure as on the disks was used as both a heating element and as a sensor and was applied on top of the layer to be investigated. It was then periodically heated and the temperature oscillation was measured with the sensor as a response, from which the thermal conductivity of the Al_2_O_3_ and hydrocarbon layer could be determined. The specific heat capacity and density were taken from the literature, Table 3.

The sensor type is a positive temperature coefficient (PTC) thermistor. The temperature dependence of the electrical resistance can be approximated by a linear function with a positive temperature coefficient $\alpha_{T}$, see Equation (8). Due to variations in the deposition and structuring process, $\alpha_{T}$ is not exactly the same for each sensor. Therefore, the thermoresistive characteristic was measured for each sensor. For this purpose, the sensor was connected to a constant current source and heated in an oven. An additional surface-mounted temperature sensor and a voltmeter were used to determine the temperature dependence of the resistance by Ohms law. It was found that the sensors describe a linear relationship in a range of 20–120 °C.

**Figure 3:**
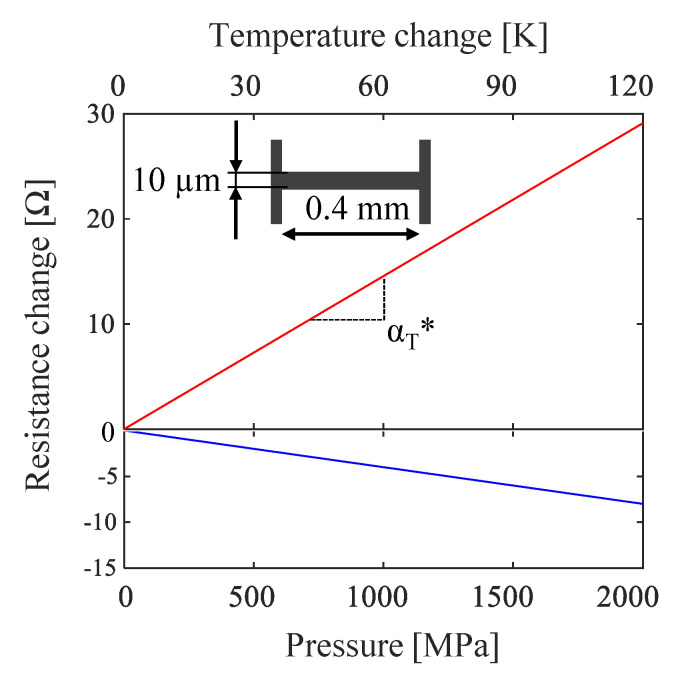
Temperature (red line) and pressure dependency (blue line) of a line sensor.

In addition to the temperature dependence of the sensor resistance, the resistance changes due to mechanical stress (piezo-resistive effect) must also be considered. While the sensor passes the contact area it is mechanically loaded which leads to a decrease of the sensor resistance. In order to analyze this effect, slip-free pretests up to 3 kN were carried out with the two-disk test rig. The change in resistance describes an integral due to the finite length of the sensor passing the contact ellipse. The absolute peak was determined as the maximum absolute value of resistance change and then plotted against the maximum pressure p_max_. A linear relationship was found. The values and dependency of temperature and pressure coefficients are shown in Table 4 and Figure 3, respectively.

In this study two designs were tested, a line and a loop design. The line design is a single straight line between the contact wires, Figure 3. That simple design allows measurements with a high spatial resolution. The loop design consists of two parallel lines, which are connected with a loop. Thereby, the relative resistance change α_T_* is higher and this will increase the temperature resolution. However, a longer sensor decreases the spatial resolution. A comparative measurement is shown in Section 3.

### 2.3. Test Procedure

**Figure 4:**
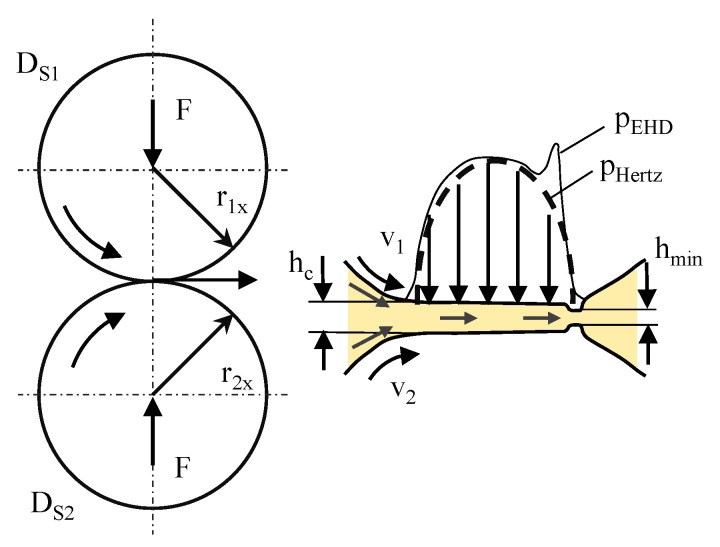
EHL.

The processes within contacts subjected to rolling are complex and efforts have been made for several years to describe them analytically and numerically in order to derive optimization potential for technical applications. The focus is particularly on the lubrication gap height h, which allows fundamental statements to be made about the wear and friction of the system. The optimization or minimization of wear and friction in lubricated tribological systems is achieved by separating the contact surfaces using hydrodynamic lubrication. Hydrodynamic lubrication describes the build-up of a lubricating film in the presence of a transport effect of the lubricant due to a surface velocity v into the decreasing gap. The theoretical extension of elastic contact partners, which describes the flattening of the contact area, refers to elastohydrodynamic lubrication. The gap can be approximately characterized with the central film thickness h_c_ or the minimum film thickness h_min_ (Figure 4) using equations according to Hamrock and Dowson depending on geometry R, velocity U, material and lubricant data G, as well as load W, Equations (10)–(11) [25].

(10) $$h_{c}=2.69\;\cdot {\;R}_{x}\;\cdot \;\frac{G^{0.53}\;\cdot {\;U}^{0.67}}{W^{0.067}}\cdot \;(1\;-\;0.61\;\cdot {\;e}^{-0.73\kappa })$$

(11) $$h_{\min}=3.63\;\cdot {\;R}_{x}\;\cdot \;\frac{G^{0.49}\;\cdot {\;U}^{0.68}}{W^{0.073}}\;\cdot \;(1\;-\;0.61\cdot e^{-0.68\kappa })$$

Corresponding to the EHL-theory, the minimum film thickness is located at a contraction in the outlet section of the contact area and mixed friction is to be expected here first. The deformation of the surface in this area can be explained by the interaction of the elastic disks and the hydrodynamics of the lubricant.

With the comparison of the film thickness to the critical film thickness h_crit_, a statement about the friction condition—mixed friction h_crit_ > h_min_ or fluid friction h_crit_ ≤ h_min_—can be made. The critical film thickness h_crit_ can be calculated analytically using the root mean squared roughness value Rq of the disk surfaces. However, this assumes a normal distribution of the surface topography, which was not present for the rough crowned disk in this study. Another approach for determining the critical film thickness is to use a discrete model of elastic/plastic solid body contact. Based on the real measured surface data (see Table 1) and the elastic-plastic material properties (see Table 2), a solid body contact curve can be calculated and the critical film thickness can in turn be derived (Table 5) [26]. The tool MicroSim was used for the calculation [27].

With the equations of Hamrock and Dowson in mind, comprehensive temperature measurements for fluid and mixed friction were carried out with the presented sensor system. With regard to technical applications, tests were run up to 1750 MPa surface pressure, 80 °C oil feed temperature, 8 m/s total speed, and 40 % slip. Surface pressure in combination with friction leads to localized failure of the coating system. Therefore, the tests were limited to 1750 MPa. With decrease of the lubricating film, the surfaces get closer and after reaching a limit lubricating film thickness, localized contact of the surface occurs. Since the contact forces are no longer transmitted completely across the lubricating film, mixed friction appears. In light of this, special demands on the wear resistance of the top protective layer are required.

In order to reach mixed friction, mainly the total speed was reduced and this effectively led to a reduced lubricating film thickness. The lubrication condition was pre-determined with the EHL equation. With these results, two sets of parameters were derived, which divide the tests into fluid and mixed friction. The well characterized reference oil FVA 3 was used as lubricant, Table 6.

According to Equation (8), the resistance change of the sensor is composed of a temperature and a pressure part. The temperature change in a rolling contact is significantly influenced by the pressure p and slip S or sliding speed v_Sli_. Within these temperature measurements, the pressure component represents a measuring error that distorts the measuring signal. To determine the temperature part from the measurement signal, the pressure part was measured in a slip-free preliminary test. It can be assumed that with the test conditions and a sliding velocity of zero there is only a negligible temperature change in the contact. Therefore, the slip-free preliminary tests were used to calculate the temperature signal from the measurement signal afterwards. To determine the pressure component, a permanent magnet was mounted on the shaft as a trigger point with a fixed position to the sensor. Despite the very precise speed control, it was necessary to manually adjust the time shift during the evaluation.

## 3. Design Study

**Figure 5:**
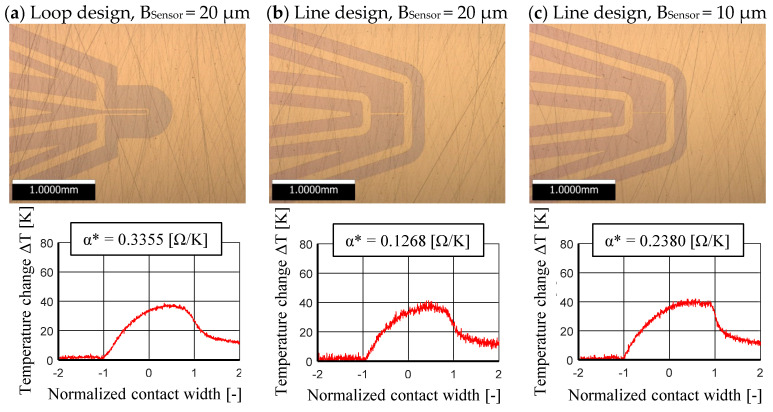
Comparison of different sensor designs at p_max_ 1500 MPa, T_Oil_ 40 °C, v_tot_ 2 m/s, and Slip 40%.

In advance to the measurements of the contact temperature during mixed and fluid friction according to Table 6 the measuring capability of a loop and line design was pre-investigated by an experimental study. For the comparison of the real sensor systems, a loop design and two straight line designs were coated on one disk, Figure 5.

The loop design (a) is characterized electrically by a high output resistance and thus with the highest α* value of the three sensors. This implies that even small temperature changes can be recorded as voltage changes and the background noise is lower. This advantage comes however with a larger sensor surface. The larger sensor surface in comparison to the line sensors leads then, as already shown above, to a decline of the spatial resolution. The line design with 20 µm sensor (b) width has the smallest output resistance of the three investigated sensor designs. This is visible for the strong noise of the measurement signal. Especially a small temperature change due to a small sliding speed would make a proper measurement more difficult. To increase the output resistance at the same sensor length, the chromium layer thickness or the sensor width can be reduced. However, the reduction of the layer thickness at a height of 200 nm is no longer suitable. On the other hand, it was possible to reduce the sensor width to 10 µm by a process improvement while maintaining a high structure quality. This improves the signal-to-noise ratio and leads to a high spatial resolution. For the further investigations, this sensor design (c) with the above described geometrical, mechanical and thermal properties was used, Section 2.2.

## 4. Test Results

**Figure 6:**
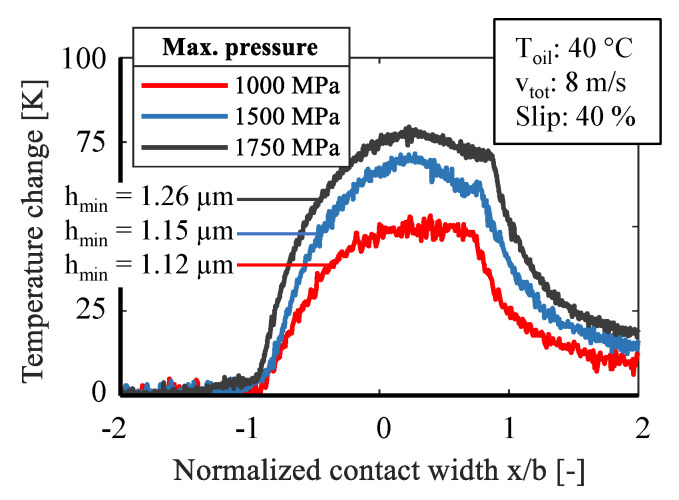
Influence of pressure at fluid friction on temperature change.

The following figures show the temperature change of the sensor while in contact and under different test conditions, Table 6. For a better comparability the contact width was normalized with the Hertzian equations. Figure 6 shows the measured temperature change of the sensor under technically demanding conditions. The surface pressure was increased from 1000 MPa to 1750 MPa while retaining the same conditions of 40 °C oil supply temperature, 8 m/s total velocity and 40 % slip. The measurement data illustrate that immediately after the sensor enters the contact area, its temperature rises and in the second part of the contact area the maximum sensor temperature is reached. As expected, the measured contact temperature increases at a higher surface pressure. Under otherwise identical conditions, a temperature increase of 50.3 K at 1000 MPa, 70.0 K at 1500 MPa, and 77.7 K at 1750 MPa surface pressure was determined. After reaching the maximum temperature, the sensor temperature drops to a lower peak. This can be explained by the EHL-theory and the minimum film thickness *h_min_* at this point. After the second peak the temperature drops with a typical cooling characteristic. The minimum film thickness differs from 1.26 µm at 1750 MPa to 1.12 µm at 1000 MPa. Therefore, fluid friction can be assumed in all three measurements.

**Figure 7:**
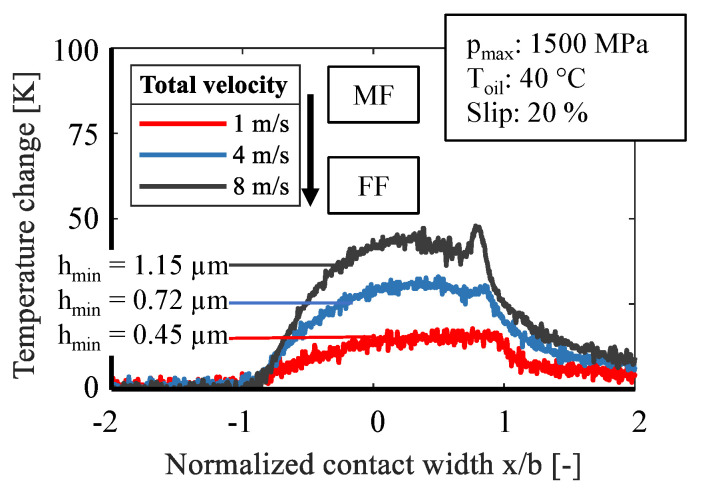
Influence of total velocity on temperature change.

Figure 7 presents measurement curves at 1500 MPa pressure, 40 °C oil supply temperature, and 20 % slip. The total velocity was increased from 1 m/s to 4 m/s and 8 m/s, respectively. On the one hand, the higher total speed results in a higher sliding speed, which leads to a higher frictional power and thereby to a higher contact temperature. On the other hand, with a higher total speed, more oil is pressed into the lubrication gap while the minimum film thickness rises from 0.45 µm at 1 m/s to 1.15 µm at 8 m/s. Hence, at a high total velocity of 8 m/s and 4 m/s fluid friction is more likely to be present, and at low total velocity of 1 m/s mixed friction is expected. The change of the friction state becomes clear by the change of the formation of the second peak. If the total speed decreases, the solid contacts increase and the second peak decreases or disappears because of the missing hydrodynamic.

**Figure 8:**
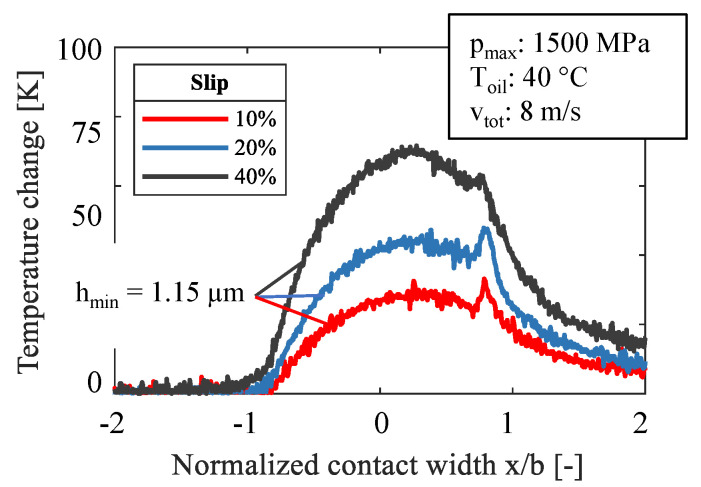
Comparison of slip at fluid friction.

**Figure 9:**
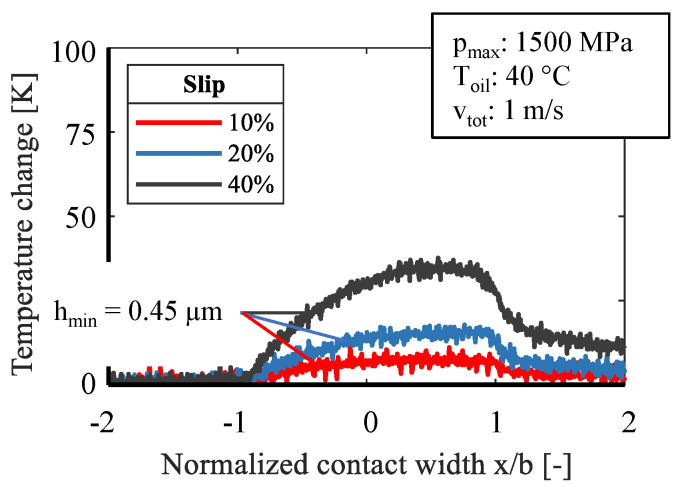
Comparison of slip at mixed friction.

Figure 8 and Figure 9 show measured data for slip values of 10%, 20%, and 40% while Figure 8 presents data for pure fluid friction and Figure 9 for severe mixed friction. The sliding speed increases at a constant total speed with the slip, which in turn leads to a higher contact temperature while the film thickness remains constant, according to the EHL-theory. This applies to both fluid and mixed friction. As already seen in Figure 6, the change of the lubrication state is strongly influenced by the total velocity. This explains the lower temperature change at mixed friction. A significant difference can be seen in the formation of the second peak at the outlet. At a high hydrodynamic speed, the contraction effects the hydrodynamic pressure and this influences the distinctive temperature rise in this zone. Meanwhile, this effect does not appear for severe mixed friction due to a lack of hydrodynamic and a large number of solid contacts.

**Figure 10:**
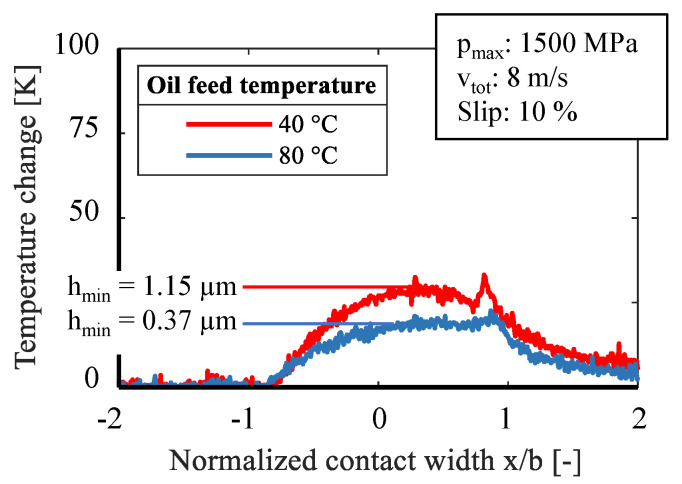
Influence of oil feed temperature on temperature change at 10% slip.

**Figure 11:**
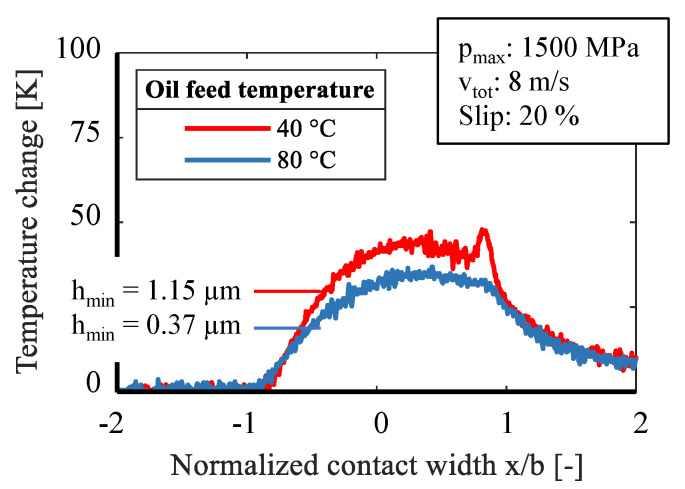
Influence of oil feed temperature on temperature change at 20% slip.

In a further series of tests, the oil feed temperature was increased from 40 °C to 80 °C under otherwise constant conditions, Figure 10 and Figure 11. The viscosity of the used reference oil changes considerably with the oil temperature [28]. Due to the lower viscosity, a thinner lubricating film is formed and mixed friction becomes more likely with a decrease of the minimum film thickness from 1.15 µm to 0.37 µm. In both figures it can be seen that with a higher oil feed temperature, the change of the sensor temperature decreases. This applies to 10% and 20% slip. Likewise, a distinctive second peak at the outlet is missing. This could be due to heavy mixed lubrication and increased solid contacts.

## 5. Discussion

The results of the presented sensor system have shown that the temperature change can be determined with a high spatial resolution for rolling contacts while mixed friction and high surface pressure occur.

The test series were carried out at first with low mechanical load, since material removal of the wear protection layer was considered. Repeated measurements, however, revealed a good reproducibility of the temperature measurements even after test series with severe mixed friction, high sliding velocity and high surface pressure.

It should be noted that the measured sensor temperature is not exactly the actual lubricating film temperature as in a steel-steel contact. Due to the lower thermal conductivity of the sensor layers and the resulting thermal barrier effect, the maximum oil temperature is located close to the coated disk surface. Despite the thin wear protection layer, the barrier effect is less distinctive to the sensor. Nevertheless, it can be assumed that with this configuration and test conditions the sensor temperature change *ΔT* is almost half the maximum lubricant film temperature change. In a further simulation study, calculations with a TEHL-model will provide more detailed information about the temperature distribution within the disks and the lubricating film.

Although chromium is used for pressure measurements [13,15] in rolling contacts, the procedure of measuring the temperature change with a chromium sensor and an additional wear protection layer proves to be successful. However, it is stated that a slip-free preliminary test is part of every temperature measurement. It was assumed that no or only a small temperature increase is present for the slip-free preliminary runs. It was also assumed that the pressure distribution of the slip-free preliminary test corresponds to tests with slip and that shear stress due to friction only has minor influence. A second sensor with a different material and consequently with a deviating pressure and temperature dependency should improve the measurements [2,3,4,9,10,11,15,17,18,21].

The lubricant film thickness as well as the pressure was not measured directly by the presented thin-film sensor. Thus, the presence of mixed friction could only be determined indirectly by EHL-theory. Even though the EHL given by Hammrock and Dowson is an accepted theory, modern TEHL-models allow more precise statements. For further simulation, the samples were thermally and mechanically characterized in detail and the micro- and macro-geometry was determined.

It can be further assumed that a higher oil feed temperature or a lower total velocity leads to a smaller lubrication film thickness, but a higher contact temperature, e.g., due to a higher slip, does not necessarily lead to a reduction of the lubrication film thickness, Figure 8, Figure 9, Figure 10 and Figure 11. This can be explained by bearing effects before and after the contact area.

## 6. Conclusions

First, three sensor designs were compared with respect to their spatial resolution and their noise behavior under constant conditions. The results are similar in quantity and quality, whereby a better spatial resolution and lower noise can be seen with a very slim line sensor. To detect effects like the second peak at the outlet of a small elliptical contact area, this sensor is suitable. The sensor can also detect the temperature change of point-like rolling contacts due to its short length properly. The spatial resolution could be further improved by a shorter line. However, with a shorter sensor the background noise increases. This effect could be compensated by reducing the sensors cross section.

A two-disk test rig was used for the measurements. The presented sensor system was applied to simple steel disks. The numerous test data show that the sensor is able to perform even under severe conditions, e.g., mixed friction and high surface pressure. A next step could be to place the sensor on components like gears or roller bearings. A lot of rolling contacts in technical applications are facing mixed friction. Thus, the thin film sensor was coated with a wear protection layer to protect the sensor material and to avoid short circuits during mixed friction. Due to the additional thermal barrier effect of the wear protection layer, the measuring data do not correspond to the oil film temperature. However, with modern calculation methods it is possible to derive the oil film temperature from the sensor temperature. Therefore, in addition to the extensive testing, a detailed sample characterization was carried out in advance to compare calculation results with test data on the basis of the sensor temperature change. To increase the performance of the sensor, an additional pressure measurement could be implemented to determine the pressure distribution under sliding. This also avoids slip-free preliminary tests with time shift problems. However, this again implies a high stress capability of the double sensor with numerous load changes. Another development could be a change of the signal transmission to wireless technology in order to reach a more application-related level.

With regard to the collected measurement data, it can be stated in general that the contact temperature is, as expected, strongly dependent on the surface pressure, the slip or sliding speed, respectively, and the state of friction.

The viscosity decreases at a higher oil supply temperature of 80 °C compared to 40 °C. This makes mixed friction due to solid contact more likely and higher friction can be assumed. That leads to higher frictional power and furthermore to an increase of the temperature change, although the contact temperature change is lower compared to an oil supply temperature of 40 °C. This illustrates the complex interactions within rolling contacts and shows the complex challenge to optimize rolling contacts within technical applications.

## Figures and Tables

**Table 1:** Macro and micro geometry.

	Macro Geometry			Micro Geometry (300 µm × 300 µm)		
DISK	D [mm]	D_Crown_ [mm]	Finishing	Sa [µm]	Sq [µm]	Sz [µm]
Sensor disk	65.00	-	Polishing	0.019	0.025	0.298
Crowned	64.96	200.04	Grinding	0.201	0.259	2.167
Sensor Disk				Crowned Disk		
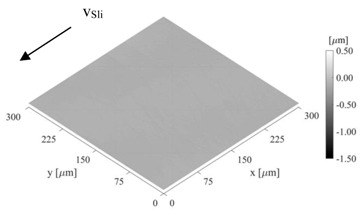				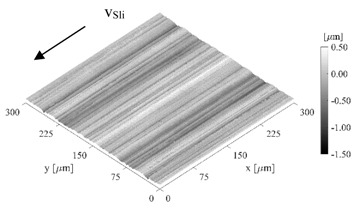		

**Table 2:** Elastic-plastic properties of the surfaces.

Material	Intendation Modul E_IT_	Martens Hardness HM
Disks 16MnCr5	300 GPa ± 10.0 GPa	7.500 GPa ± 0.210 GPa
a-C:H:Si:O Coating	38 GPa ± 2.7 GPa	2.460 GPa ± 0.165 GPa

**Table 3:** Thermal material properties.

Coating	Material	Thickness µm	Thermal Conductivity W/(m·K)	Thermal Capacity J/(kg·K)	Density kg/m^3^
Isolation	Al_2_O_3_	4.6	1.62	850	1632
Sensor	Cr	0.2	-	-	-
Wear protection	a-C:H:Si:O	2.5	0.63	700	2900
Base material	Steel (16MnCr5)	-	44	431	7760

**Table 4:** Elastic-plastic properties of the surfaces.

Material	Temperature Coefficient α_T_ 1/K	Pressure Coefficient α_P_ 1/MPa
Chrome	8.3 × 10^−4^	1.5 × 10^−5^

**Table 5:** Critical film thickness.

Parameter	Value
Critical film thickness h_crit_ in µm	0.716

**Table 6:** Test conditions for slip.

Parameter	Fluid Friction	Mixed Friction
Pressure p_max_ in MPa	1000/1500/1750 *	1500
Oil feed temperature T_Oil_ in °C	40	80
Velocity v_tot_ in m/s	4/8	8
Slip S in %	10/20/40	
Lubricant	Reference Oil FVA 3	

* Only at 40 °C oil feed temperature.

## Data Availability

Not applicable.

## Nomenclature

Contact deformation	
A_Hertz_	Hertzian contact area
a, b	Semi-axis of the elliptical contact area
x, y	Cartesian coordinates
F	Normal force
p, p_max_	Pressure
D_S1_, D_S2_, D_Crown_	Disk diameter
ρ	Curvature
E_Red_, E_IT_	Reduced E-Modul, Indentation E-Modul
c_a_, c_b_	Correction term
EHL-theory	
v_Tot_, v_1_, v_2,_ v_Sli_	Surface velocity
h_c_, h_min_	Central and minimal film thickness
R, G, U, W	Geometry-, material-, velocity-, load-parameter
κ	Correction term
S	Slip
Sensor system	
ΔR, R_0_	Resistance change, output resistance
ΔT	Temperature change
Δp	Pressure change
α_T_	Temperature coefficient
α_P_	Pressure coefficient
IConst	Constant current
U	Electrical voltage
